# EPID dosimetry for pretreatment quality assurance with two commercial systems

**DOI:** 10.1120/jacmp.v13i4.3736

**Published:** 2012-07-05

**Authors:** Daniel W. Bailey, Lalith Kumaraswamy, Mohammad Bakhtiari, Harish K. Malhotra, Matthew B. Podgorsak

**Affiliations:** ^1^ Department of Radiation Medicine Roswell Park Cancer Institute Buffalo; ^2^ Department of Physics State University of New York at Buffalo Buffalo; ^3^ Department of Physiology and Biophysics State University of New York at Buffalo Buffalo NY

**Keywords:** EPID, quality assurance, IMRT QA, VMAT QA, portal dosimetry

## Abstract

This study compares the EPID dosimetry algorithms of two commercial systems for pretreatment QA, and analyzes dosimetric measurements made with each system alongside the results obtained with a standard diode array. 126 IMRT fields are examined with both EPID dosimetry systems (EPIDose by Sun Nuclear Corporation, Melbourne FL, and Portal Dosimetry by Varian Medical Systems, Palo Alto CA) and the diode array, MapCHECK (also by Sun Nuclear Corporation). Twenty‐six VMAT arcs of varying modulation complexity are examined with the EPIDose and MapCHECK systems. Optimization and commissioning testing of the EPIDose physics model is detailed. Each EPID IMRT QA system is tested for sensitivity to critical TPS beam model errors. Absolute dose gamma evaluation (3%, 3 mm, 10% threshold, global normalization to the maximum measured dose) yields similar results (within 1%–2%) for all three dosimetry modalities, except in the case of off‐axis breast tangents. For these off‐axis fields, the Portal Dosimetry system does not adequately model EPID response, though a previously‐published correction algorithm improves performance. Both MapCHECK and EPIDose are found to yield good results for VMAT QA, though limitations are discussed. Both the Portal Dosimetry and EPIDose algorithms, though distinctly different, yield similar results for the majority of clinical IMRT cases, in close agreement with a standard diode array. Portal dose image prediction may overlook errors in beam modeling beyond the calculation of the actual fluence, while MapCHECK and EPIDose include verification of the dose calculation algorithm, albeit in simplified phantom conditions (and with limited data density in the case of the MapCHECK detector). Unlike the commercial Portal Dosimetry package, the EPIDose algorithm (when sufficiently optimized) allows accurate analysis of EPID response for off‐axis, asymmetric fields, and for orthogonal VMAT QA. Other forms of QA are necessary to supplement the limitations of the Portal Vision Dosimetry system.

PACS numbers: 87.53.Bn, 87.53.Jw, 87.53.Kn, 87.55.Qr, 87.56.Fc, 87.57.uq

## I. INTRODUCTION

Intensity‐modulated radiation therapy (IMRT) has become a standard modality for delivering highly conformal dose distributions compared to 3D conformal techniques. As an alternative to IMRT delivery, volumetric‐modulated arc therapy (VMAT) is a relatively new dose delivery technique allowing delivery of highly conformal dose distributions in a shorter period of time and with fewer monitor units as compared to traditional IMRT.^(^ ^1^ ^,^ ^2^ ^)^ RapidArc (Varian Medical Systems, Palo Alto CA) is one commercially available method of delivering VMAT treatments, in which the dose distribution is ideally delivered in one arc with 177 control points, each linking a specific MLC position to a specific gantry angle.^(^ ^3^ ^–^ ^5^ ^)^ Because of the high complexity and uniqueness of IMRT and VMAT treatment plans, patient‐specific pretreatment quality assurance (QA) is generally considered a necessary prerequisite to patient treatment.^(^ ^6^ ^,^ ^7^ ^)^ Much interest has been shown in the use of electronic portal imaging devices (EPIDs) for such dosimetry measurements.^(^ ^8^ ^–^ ^12^ ^)^ High contrast, large detector density, large detecting surfaces, linear response to radiation dose, and efficient online capabilities make EPIDs tempting candidates for IMRT QA.^(^ ^13^ ^–^ ^16^ ^)^ At the same time, however, high‐Z component materials render EPIDs far from water‐equivalent. Consequently, a number of institutions and vendors have produced algorithms to either predict calibrated EPID response, or to convert calibrated EPID response into a simulated dose plane, such that EPID images can be used to verify the calculation and delivery of IMRT fields.^(^ ^15^ ^,^ ^17^ ^–^ ^22^ ^)^ In this study, we analyze and optimize the physics modeling of a recently developed EPID dosimetry algorithm — EPIDose (Sun Nuclear Corporation, Melbourne, FL) — and compare its performance to the Portal Dosimetry system (Varian Medical Systems, Palo Alto, CA). Each EPID dosimetry system provides a different approach to commissioning and calculation, and these differences are analyzed. IMRT QA results for clinical plans of varying modulation intensity are compared between the Varian and Sun Nuclear EPID dosimetry systems, and in addition compared to similar results from a standard planar diode array, MapCHECK (Sun Nuclear Corporation). Specifically, the IMRT QA process is examined for highly complex fluences and off‐axis, asymmetric fluences. Further, each system is tested for its ability to catch two critical TPS dose calculation errors. Lastly, we explore and analyze the intriguing possibility of performing EPID‐based pretreatment QA for VMAT treatments using the EPIDose system.

## II. MATERIALS AND METHODS

All EPID images analyzed in this study were acquired with an amorphous silicon (aSi), indirect‐detection EPID (Varian PortalVision aS1000) coupled to a 6 MV linear accelerator (Varian Trilogy with 120‐leaf Varian Millennium multileaf collimator (MLC)) via the Portal Vision Exact Arm (a robotic arm, attached directly to the linear accelerator (linac), that is remotely positioned with high accuracy and reproducibility^(^ ^23^ ^)^. The PortalVision aS1000 flat‐panel EPID has a $40\times 30\;{\text{cm}}^{2}$ detecting surface with a matrix of 1024× 768 pixels (0.392 mm pixel pitch). All IMRT EPID images were acquired at an SDD of 105 cm with no additional build up, with gantry and collimator at zero degrees (unless otherwise noted below). VMAT EPID images were acquired with the gantry in rotation, while the EPID itself was static with respect to the gantry. The linac beam symmetry and output (among other dosimetric parameters) were verified daily (via morning check device), and more rigorously verified on a monthly basis (via diode array and ionization chamber).

### A. Image acquisition for portal dosimetry

For image acquisition with Varian PortalVision and analysis with Portal Dosimetry, the EPID was calibrated according to the vendor's specifications, with dark field (DF), flood field (FF), and dose scaling calibrations performed each day of measurement. EPID response was scaled such that 1 Calibrated Unit (CU) corresponds to 100 MU delivered by a $10\times 10\;{\text{cm}}^{2}$ open field at 100 cm SDD. Unless otherwise stated, the diagonal profile correction (used to scale the off‐axis pixel response after FF flattening) was performed as recommended by Varian: the beam intensity profile was measured at $d_{\max}$ in water for a $40\times 40\;{\text{cm}}^{2}$ open field. This profile correction is applied upon each absolute dose calibration. Dosimetric analysis of PortalVision dose images was performed via Varian Eclipse Version 8.6, including Portal Dosimetry Version 8.2.24. All IMRT fields were delivered to the linac treatment console via the MOSAIQ record and verify system (Impac MOSAIQ Version 1.6, Elekta Oncology Systems, Norcross, GA).

### B. Image acquisition for EPIDose

EPIDose employs DICOM RT EPID response images and converts them into dose planes for comparison to similar TPS calculated planes. There are several methods available for Varian PortalVision users to acquire these integrated images. Whichever option is selected, the acquisition method must be consistent between EPIDose commissioning measurements and all subsequent image acquisitions. For the PortalVision system, Sun Nuclear suggests collecting EPID images within the Acquisition Module (AM) Maintenance software which accompanies PortalVision. However, for clinical QA, this method bypasses the record and verify system, such that there is no automatic recording of the QA delivery in the patient's electronic medical record. Instead, the desired radiotherapy plan must be exported from the TPS and transitioned to the treatment console manually (i.e., by network or portable drive), and the machine parameters must be manually loaded with the linac in service mode. Consequently, this delivery method audits the treatment plan, but not the actual fluence that was transferred to the record and verify system for patient treatment.

As an alternative, EPID images can be acquired through the record and verify system with the linac in clinical mode, thereby verifying the actual treatment fields. As with images collected via the AM Maintenance interface, this method automatically applies the most recent DF, FF, and CU calibration (along with the diagonal profile correction). As discussed in the Materials and Methods Section II. C below, the EPIDose commissioning process correlates EPID response with similar MapCHECK dose measurements in order to convert EPID response to dose in water. Thus, the CU scaling and diagonal profile correction, each vital to the Varian Portal Dosimetry system, are not required for images used with EPIDose. For example, as long as the chosen dose scaling value (typically unity for 100 MU delivered to the EPID at 100 cm SDD) is constant in all EPID images acquired for EPIDose, this number is arbitrary to the EPIDose system. Given that the diagonal profile correction recommended by Varian is approximate and problematic,^(^ ^24^ ^–^ ^25^ ^)^ we developed a new method of acquiring PortalVision EPID images for EPIDose conversion that effectively bypasses the diagonal profile correction. The PortalVision EPID was calibrated with DF, FF, and CU calibrations as outlined in Materials and Methods Section II. A, but the diagonal beam profile was replaced with a modified text file indicating a perfectly flat beam (i.e., all off‐axis correction factors being unity). Thus, for all subsequent EPID images, the off‐axis diagonal profile correction has no scaling effect whatsoever, yielding EPID images that are simply DF‐ and FF‐corrected. In this way, the EPIDose physics modeling process compares flattened EPID response images to similar MapCHECK dose measurements, allowing the EPIDose commissioning algorithm to produce its own two‐dimensional model of off‐axis EPID response without using the Varian off‐axis approximations. It should be noted that this choice of image acquisition was preferred only from the perspective of wanting to entirely avoid the physically problematic Varian diagonal profile correction, but the EPIDose algorithm is robust enough to compensate for these approximations and produce the same clinical results even if the standard Varian calibration is employed. It is simply vital that the exact same acquisition method is used for both commissioning EPIDose and all subsequent acquisitions for clinical plans.

### C. Optimizing the EPIDose algorithm

Both the Portal Dosimetry prediction algorithm^(^ ^21^ ^)^ and the EPIDose calculation model^(^ ^17^ ^)^ have been discussed in previous studies. The two approaches to EPID dosimetry are markedly different: Portal Dosimetry provides a prediction algorithm to model the response of the detector, while EPIDose provides a calculation algorithm to convert from detector response to dose in water. In addition to the fact that Varian's algorithm compares calibrated EPID images to predicted images while Sun Nuclear's algorithm compares EPID calculated dose to TPS calculated dose, two other differences between the two systems further affect the results of this study — the EPIDose optimization process and the EPIDose correction for EPID response to MLC transmitted radiation, both of which are discussed in detail below.

The EPIDose software converts EPID images into dose planes via a four‐step algorithm^(^ ^17^ ^)^ which first converts EPID images to relative dose and then scales the converted image to absolute dose. The four steps are: 1) image back‐projection accounting for divergence of the beam between the source‐detector‐distance (SDD) and desired source‐dose plane‐distance (SPD); 2) output factor matrix accounting for variation in EPID response to field size (i.e., effective field size of each segment for IMRT fields) and MLC transmission; 3) dose redistribution via a point‐spread kernel which converts measured EPID response to relative dose response at depth in water; and 4) a two‐dimensional conversion from relative to absolute dose response for each pixel. All beam data is acquired by measuring commissioning fields with the MapCHECK diode array. Thus, EPIDose‐calculated dose planes can be directly compared to TPS calculated dose planes (at specified depth in water), providing an independent additional check of the actual TPS dose calculation algorithm. The EPIDose physics modeling process allows optimization of the EPID physics model in order to best match similar EPIDose‐calculated and MapCHECK‐measured dose planes. It is most important to note that this process is not used to optimize agreement between EPIDose and TPS calculated planes. However by comparing to MapCHECK, a standard of dose plane measurement independent of the TPS, the optimization process ensures that the EPIDose calculated dose plane is an accurate reflection of the actual dose delivered by each fluence. Since the commissioning and validation process of the EPIDose system relies heavily on the MapCHECK device, this process assumes that the MapCHECK device itself has been properly calibrated and validated for absolute dosimetry measurement of intensity modulated fields.

The EPIDose optimization process is accomplished in three stages, each detailed below: optimizing the field‐size correction factors, optimizing the relative dose distribution kernel, and finally optimizing EPID response to MLC transmission. Throughout the EPIDose physics model optimization process, EPIDose calculated dose planes were compared to similar MapCHECK measured dose planes to track improvement of the physics model — fields used for these commissioning comparisons were $10\times 10\;{\text{cm}}^{2}$ and $20\times 20\;{\text{cm}}^{2}$ open fields, and three fields minimum from each of the clinical categories analyzed in this study (detailed below). EPIDose absolute dose was quantitatively compared to MapCHECK dose via distance‐to‐agreement (DTA) and percent difference composite analysis with parameters of 2 mm, 2%, and a 10% dose threshold (with percent differences normalized to maximum planned dose).

**Figure 1:**
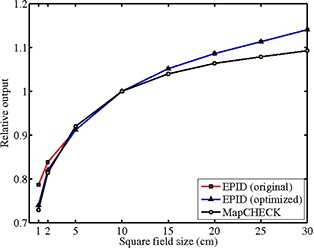
EPIDose and MapCHECK output factors: EPID original, EPID optimized, and MapCHECK for 1× 1, 2× 2, 5× 5, 10× 10, 15× 15, 20× 20, and $25\times 25\;{\text{cm}}^{2}$ square fields, normalized to the $10\times 10\;{\text{cm}}^{2}$ output.

The field size correction factors account for the fact that EPID response is not the same as water‐phantom response to the same machine and beam conditions.^(^ ^13^ ^,^ ^14^ ^,^ ^17^ ^)^ These corrections are calculated from the ratio of dose response (i.e., MapCHECK) and EPID response to the same setup and number of MU for various field sizes, normalized to the $10\times 10\;{\text{cm}}^{2}$ response.^(^ ^17^ ^)^ Because the commissioning measurements were acquired with jaw‐blocked fields while each IMRT segment is a transition between two MLC‐blocked beam shapes, the relative output values measured with the EPID were used as initial guesses rather than strict modeling parameters. Figure 1 shows three sets of relative output factors for square fields of sizes 1× 1, 2× 2, 5× 5, 10× 10, 15× 15, 20× 20, and $25\times 25\;{\text{cm}}^{2}$ — EPID measured, MapCHECK measured, and finally EPIDose optimized. The $1\times 1\;{\text{cm}}^{2}$ and $2\times 2\;{\text{cm}}^{2}$ values were important in optimizing the EPIDose model for complex fluences. These smaller output factors affect the highly modulated fluences (e.g., head and neck (H&N)) more substantially than the less complex fluences (e.g., prostate and breast) due to the fact that the effective (i.e., MLC‐blocked) field size per control point is generally smaller for more complex fields. Thus, the $1\times 1\;{\text{cm}}^{2}$ and $2\times 2\;{\text{cm}}^{2}$ initial EPID response values were varied as fitting parameters in increments of 0.01 until optimal results were achieved between EPIDose‐calculated and MapCHECK‐measured fields. According to the EPIDose vs. MapCHECK comparison performed for all the IMRT fields used in commissioning EPIDose for this study, no optimization was needed for the EPID field size factors larger than $2\times 2\;{\text{cm}}^{2}$.

**Figure 2:**
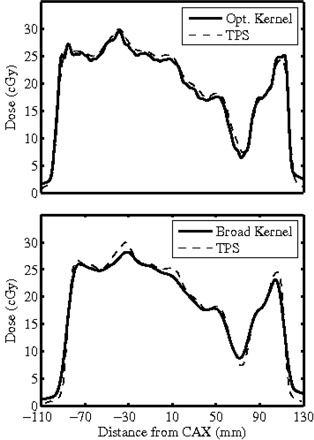
EPIDose redistribution kernel, which is analogous to a point spread function or scatter kernel, converts EPID scatter to dose scatter. Broadening the dose kernel effectively flattens the EPIDose calculation, raising dose valleys and lowering dose peaks.

Similarly, the relative dose distribution kernel used to convert EPID response to dose at QA depth within a homogeneous water phantom was manually changed in small increments, beginning with the vendor's suggested default kernel, to further optimize the EPIDose calculations. As the kernel was altered, EPIDose calculations were compared to similar MapCHECK measurements until agreement was optimal, as measured by composite analysis of 2% dose difference and 2 mm DTA. Changing the kernel most profoundly affects dose peaks and valleys (e.g., a broader kernel raises the dose in valleys and lowers the dose in peaks). Figure 2 shows an in‐plane profile along the central axis of the EPID for a H&N fluence, comparing EPIDose to TPS dose: the upper figure shows EPIDose calculated with the optimized dose kernel, while the lower figure shows the same profile for an EPIDose calculation with a kernel that is too broad. Notice that in the lower figure, the profile is effectively flattened, with noticeably lower dose peaks and higher dose valleys than those resulting from the optimized kernel.

Finally, the EPIDose software also allows optimization of a correction parameter that accounts for EPID response to beam spectral variation due to MLC transmission.^(^ ^26^ ^)^ A single correction factor, termed the “Dose/EPID for MLC Transmission,” is multiplicatively applied to the response of the pixels in those regions covered by the MLC leaves for each segment in the treatment plan. To define these covered regions, the EPIDose software prompts the user to supply either the MLC file or the RTP‐DICOM file corresponding to the fluence that produced the EPID image. This solution serves as an approximation of the actual MLC positions during each segment of the IMRT delivery — if individual leaves behave unexpectedly, some amount of uncertainty might be incorporated into the calculation. In order to optimize the “Dose/EPID for MLC Transmission” factor to match the specific acquisition LINAC/EPID system, a number of EPIDose physics models were created that differed only by the value of “Dose/EPID for MLC Transmission” factor, which was altered from 0.85 to 1.0 using 0.01 increments. EPIDose‐calculated planes for each of these models were compared to similar MapCHECK dose planes to find the “Dose/EPID for MLC Transmission” value achieving optimum agreement between EPIDose‐calculated and MapCHECK‐measured dose. For our particular LINAC/EPID combination, an MLC transmission factor of 0.95 was found to be optimal, yielding the highest conformity between EPIDose calculation and MapCHECK measurement for virtually all fields tested. The MLC transmission correction factor operates on every individual fluence in a unique way, depending on the positions of all MLC leaves for each segment. Highly modulated fields are impacted the most by this correction, since larger portions of the field area are covered by the MLC throughout larger portions of beam‐on time as compared to simpler fluences. Consequently, optimizing the “Dose/EPID for MLC Transmission” value affects H&N cases more substantially than prostate and breast tangent cases which are modulated comparatively very little.

For example, 14 H&N IMRT fields yielded an average pass rate of 97.9% for EPIDose vs. MapCHECK with DTA analysis of 2%, 2 mm, whether the “Dose/EPID for MLC Transmission” factor was set to 0.95 or 1.0 (i.e., no correction). However, with the tolerances lowered to 1%, 1 mm, the same fields yielded average pass rates of 86.5% and 81.2% for “Dose/EPID for MLC Transmission” factors of 0.95 and 1.0, respectively. On the other hand, when 10 prostate IMRT fields were analyzed in the same way, 0.95 and 1.0 “Dose/EPID for MLC Transmission” factors yielded the same average pass rate of 99.5% for 2%, 2 mm tolerances. When the tolerances were restricted to 1%, 1 mm, the average pass rates were 99.1% and 98.7% for “Dose/EPID for MLC Transmission” factors of 0.95 and 1.0, respectively, demonstrating that optimizing the MLC transmission correction yields more improvement for highly modulated fields than for fields with relatively low modulation complexity.

### D. Planar dose acquisition with MapCHECK

For this study, the MapCHECK diode array was employed both to commission and optimize the EPIDose physics modeling algorithm, and as the standard for evaluating the performance of both Portal Dosimetry and EPIDose.^(^ ^9^ ^,^ ^27^ ^–^ ^29^ ^)^ For IMRT measurements, the MapCHECK device was positioned at 100 cm SDD with 3 cm buildup (for a total water‐equivalent depth of 5 cm). For VMAT measurements, in order to most closely mimic the VMAT delivery conditions for the EPID, the MapCHECK device was placed in the Isocentric Mounting Fixture (IMF by Sun Nuclear Corporation) such that the diode array rotates with the gantry, always orthogonal to the beam. The device was calibrated for absolute dose using the vendor's procedures on each measurement day (to match the response of ion chamber in water), and the array calibration was performed at the beginning of the 6‐month interval in which measurements were acquired. Previous to this study, the MapCHECK device has been used extensively in our clinic to measure absolute dose delivery of IMRT and VMAT fields, while comparisons of calibrated MapCHECK response to measurements made with ionization chambers and radiographic film have shown the diode array to be a highly accurate and reliable dosimeter for these types of measurements.

### E. IMRT and VMAT plans

All test plans for this study were planned via Varian Eclipse Version 8.6 for 6 MV photons at either 400 or 600 MU/min for IMRT plans (DMLC) or the highest dose rate allowable for VMAT plans via RapidArc (i.e., 600 MU/min, though this number varies greatly during delivery to allow positioning of the gantry and MLC leaves). Previous studies demonstrate the effectiveness of both Varian and Sun Nuclear commercial EPID IMRT QA algorithms for prostate IMRT fluences,^(^ ^8^ ^,^ ^9^ ^,^ ^17^ ^,^ ^24^ ^)^ so only 10 prostate fluences were tested in this study, mainly for verifying accuracy in commissioning each QA system. The remaining plans examined in this study fall into four categories: forward‐planned electronic compensation (eComp) breast tangents, inverse‐planned IMRT H&N, inverse‐planned VMAT H&N, and inverse‐planned VMAT prostate. A total of 152 fields were tested. Table 1 shows the number of fields studied for each clinical category. The eComp breast cases subdivide into two categories: two‐field tangents (asymmetric, centrally located on EPID), and tangents from a three‐field monoisocentric technique that are asymmetric and off‐axis with respect to the center of the EPID, requiring collimator rotation to fit within the EPID surface at 105 cm SDD.

**Table 1:** Clinical fields examined in this study.

*Category*	*Number of Fields*
Prostate IMRT	10
Breast eComp	14 (2‐Field Tangents) 16 (3‐Field Tangents)
Head&Neck IMRT	86
Prostate VMAT	14
Head&Neck VMAT	12
TOTAL	152

### F. Testing sensitivity to TPS commissioning errors

The EPIDose and MapCHECK QA methods compare measured (or converted) dose planes to calculated dose planes in water, such that the TPS dose calculation is independently audited for errors in the dose calculation algorithm. However, in the Portal Dosimetry QA system, calibrated EPID images are compared directly to predicted images from the Portal Dose Image Prediction (PDIP) algorithm. The PDIP algorithm utilizes the actual fluence and certain commissioning measurements acquired with the EPID to calculate a predicted image rather than a calculated dose plane. So, to test the sensitivity of these QA systems to commissioning errors in the TPS, the 6 MV beam model of the Trilogy linac was tweaked to induce an error (using Pencil Beam Convolution 8.114). In this case, the dose rate table calibration (MU/Gy, measured via ionization chamber in water during linac commissioning, using a $10\times 10\;{\text{cm}}^{2}$ open field at 5 cm depth, 95 cm SSD) was decreased by about 5%, from 105.588 MU/Gy to 100.000 MU/Gy. For five prostate fields, the treatment plan was reoptimized, dose was recalculated, and new verification plans were created (i.e., dose planes in water for comparison to EPIDose/MapCHECK, and portal dose image predictions for comparison to calibrated Portal Vision measurements). The original fluences were measured first with all three QA systems (each calibrated immediately before data acquisition) and compared to the original verification plans; then the replanned fluences (with induced error in beam model) were measured with all three systems and compared to the new verification plans.

As a second, more IMRT‐specific test of TPS error sensitivity, the TPS linac model previously used to calculate all plans was copied and the 6 MV pencil beam convolution algorithm was recommissioned using Varian Golden Beam Data (GBD) profiles (acquired from the vendor for the Varian Trilogy accelerator, dated November 2009). Similarly, the GBD intensity profile was used to recommission the portal dose prediction model. The vendor‐supplied beam data does not exactly match the actual beam data measured with small ion chamber during the commissioning of our specific linac and, furthermore, a previous study showed that the GBD is particularly problematic for IMRT fields due to inadequate penumbra modeling.^(^ ^30^ ^)^ To test which of the IMRT QA modalities in this study would catch these errors, ten prostate IMRT fluences were used to calculate verification dose planes with both the original beam model and the modified beam model, as well as verification portal dose predictions with each model. Finally, dose planes were measured for these fluences with MapCHECK and EPIDose, and portal dose images were similarly acquired with PortalVision. Measured planes were compared to the verification planes from each beam model via gamma analysis and dose line profiles.

## III. RESULTS & DISCUSSION

### A. EPIDose physics model optimization

**Figure 3:**
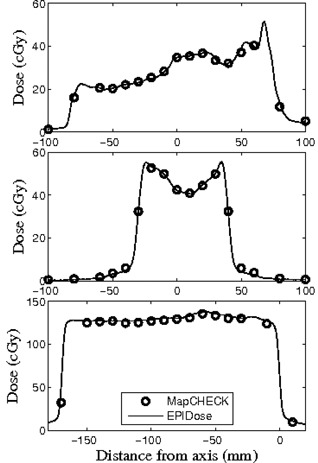
EPIDose optimization results of EPIDose‐calculated and MapCHECK‐measured response for H&N (top), prostate (middle), and monoisosentric breast tangent (bottom) plans.

With the optimization of the EPIDose physics model for our linac/EPID system complete, agreement was achieved for plans of a broad range of complexities, comparing EPIDose‐calculated to MapCHECK‐measured absolute dose via line profiles and composite dose distribution analysis. Figure 3 shows dose line profiles through the CAX for three cases used in optimizing EPIDose — one H&N dose profile (top), one prostate dose profile (middle), and one breast tangent dose profile (bottom, from a fluence extending nearly 20 cm off‐axis). DTA and percent difference composite analysis (2%, 2 mm, 10% threshold, and global normalization to maximum measured dose) for these fields yields pass rates of 99.1%, 100.0%, and 98.7%, respectively, for EPIDose vs. MapCHECK absolute dose. This extent of agreement between the EPIDose physics model and respective MapCHECK measurements gives confidence that the optimization process was successful.

### B. Differences between MapCHECK and EPIDose pass rates

**Figure 4:**
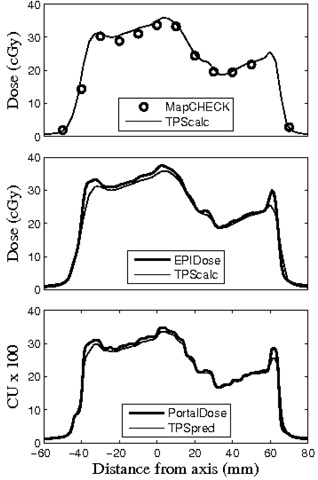
Vertical dose line profiles for the same prostate case utilized in Fig. 3: MapCHECK vs. TPS calculated (top), EPIDose vs. TPS calculated (middle), and Portal Dosimetry vs. TPS predicted (bottom).

**Figure 5:**
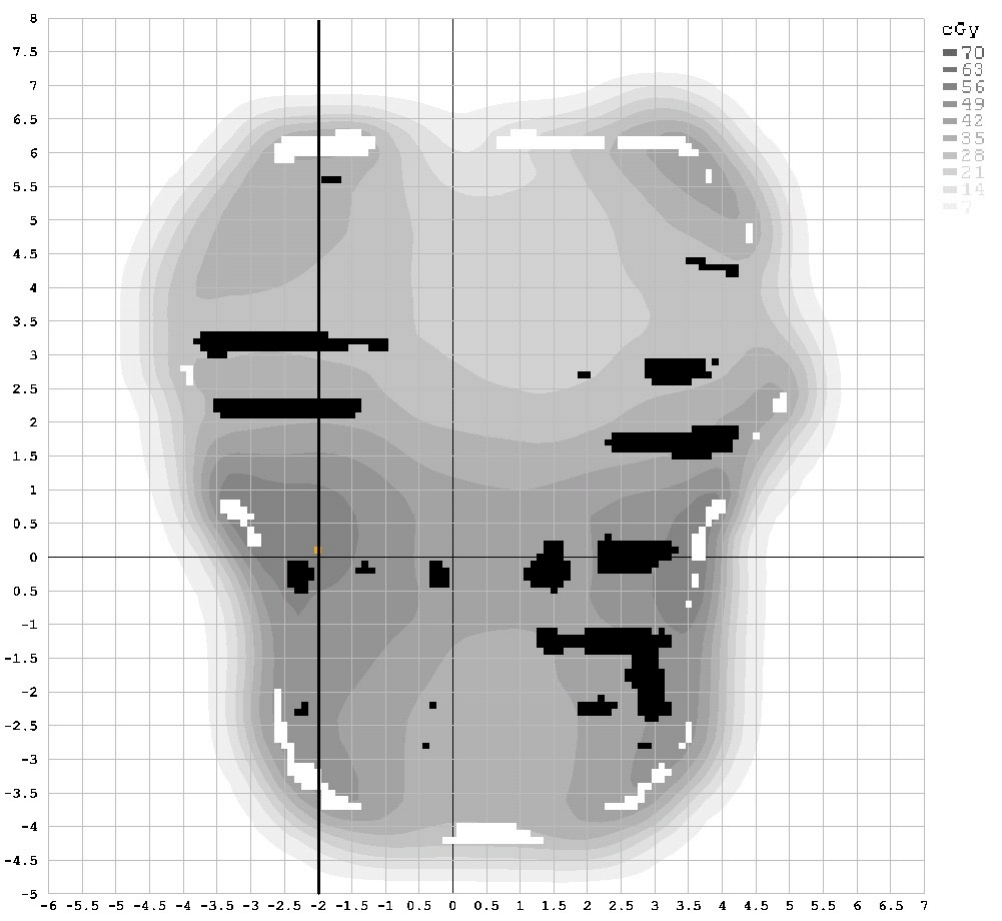
The same prostate field examined for Fig. 3 (middle), showing the vertical dose profile examined in Fig. 4, and hot (white) and cold (black) spots identified by DTA comparison to the TPS (2%, 2 mm criteria).

It must be pointed out from Fig. 3 that one should not expect exactly the same pass rate results when comparing EPIDose to the calculations of the TPS as result from MapCHECK measurement of the same field, even though the EPIDose physics model is optimized to match MapCHECK absolute dose measurements. Both the EPIDose and TPS dose planes have very high data density compared to the more discrete MapCHECK diode‐measured planes. As is clearly seen in all three examples in Fig. 3, regions with steep dose gradients may be missed by the MapCHECK diodes entirely, though much data is acquired in all regions with the high‐density pixel array of the EPID. Thus, variation is highly plausible between the respective pass rates of MapCHECK and EPIDose for the same delivery. For example, Fig. 4 shows the same dose line profile for the prostate field used in Fig. 3, this time with MapCHECK vs. TPS calculated (top), EPIDose vs. TPS calculated (middle), and Portal Dosimetry vs. TPS predicted (bottom). Meanwhile, Fig. 5 shows this dose plane, with vertical dose line profile shown in black, and hot (white) and cold (black) spots as compared to the TPS calculated plane (2%, 2 mm composite analysis). MapCHECK and EPIDose response are measured in cGy while Portal Dosimetry response is measured in CU. Notice that MapCHECK diodes are rare in the penumbral regions of this field, as demonstrated by the diodes represented in the MapCHECK dose profile. However, both EPIDose and Portal Dosimetry agree that in these very penumbral regions the delivered dose is higher than calculated and predicted by the TPS. Due to similar inconsistencies between EPIDose and TPS that are not apparent with MapCHECK analysis, composite analysis (2%, 2 mm, 10% threshold) of this field yields a pass rate of 96.4% for MapCHECK but only 92.7% for EPIDose. Such differences are not so apparent with the more typical and lenient clinical parameters of gamma evaluation^(^ ^31^ ^)^ with 3%, 3 mm tolerances, and global normalization to the maximum measured dose: 100.0% data points pass for MapCHECK and 99.3% points pass for EPIDose.

### C. EPID IMRT QA

After the EPIDose optimization process was complete and verified, the analysis of all IMRT clinical fields from H&N plans and prostate plans showed excellent agreement between MapCHECK, EPIDose, and Portal Dosimetry (unmodified). Measured (or converted) dose planes were compared to respective TPS dose calculations (or portal dose predictions) via gamma evaluation with parameters of 3%, 3 mm, 10% dose threshold (effectively limiting the evaluation to within the collimator jaws), and global normalization to the maximum measured dose. For MapCHECK measurements vs. TPS, the MapCHECK “Measurement Uncertainty” function was not turned on. Table 2 gives a breakdown of the gamma analysis results for the 10 prostate and 86 H&N fields. Numerical values represent the mean, standard deviation, minimum, and maximum of pass rates for gamma evaluation of all fields in a particular category. According to this data, MapCHECK, EPIDose, and Portal Dosimetry yield results similar to within approximately 2%, judging by the average of the pass rates of all IMRT plans (at 3%, 3 mm).

**Table 2:** Prostate and H&N IMRT analysis data.

	*Statistics*	*MapCHECK vs. TPS calc.*	*PV vs. TPS pred.*	*EPIDose vs. TPS calc.*
Prostate	mean	99.9	97.8	98.6
(10 fields)	std dev	0.03	1.0	1.0
	min	99.3	95.9	96.1
	max	100.	99.0	99.5
H&N	mean	97.8	98.4	97.7
(86 fields)	std dev	1.69	1.17	2.06
	min	91.9	93.8	90.5
	max	100.	99.9	99.9

Contrastingly, the analysis of the 30 eComp breast tangent fields does not yield such agreement between all dosimetry modalities. Table 3 presents the breakdown of the gamma analysis results for the 30 breast tangent fields subdivided into two categories, as discussed in Section II.E. In agreement with the literature,^(^ ^24^ ^,^ ^26^ ^)^ and as compared to the performance of MapCHECK, the Portal Dosimetry system does not accurately predict the EPID response to these off‐axis, asymmetric fields, and the effect worsens as distance from the CAX increases. The gamma evaluation results for these fields analyzed with Portal Dosimetry are both substantially lower and more erratic (i.e., higher standard deviation) than the results from similar analysis with the MapCHECK diode array. However, the EPIDose system performs much more closely to the MapCHECK array for measurement and analysis of these same fields, finding agreement to within 1% with MapCHECK, judging by the average of pass rates for all breast tangent plans. It should be noted from Column 5 of Table 3 that the Portal Dosimetry results from the 30 eComp fields are substantially improved by the modification suggested by Bailey et al.,^(^ ^24^ ^)^ though less so in the case of the two‐field plans. (This correction algorithm was only used to acquire additional images for these 30 fields, and only after portal dose images were first acquired according to the vendor's suggested procedures.) That work suggests a modification to the integrated acquisition calibration procedure of PortalVision and (as published) only takes effect beyond 10 cm distance from the CAX. In the case of the three‐field plans (delivered at collimator angle 90°), which extend 15–20 cm from the CAX, the correction has much more impact than on the two‐field tangents (delivered at collimator angle zero) which extend only 9–12 cm from the CAX. Even within the central 10 cm of the EPID, the Portal Dosimetry predicted values are higher for these fields than measured with PortalVision as calibrated by the vendor's unmodified specifications, and so the values in Column 5 of Table 3 may be improved even more by extending the correction algorithm back from 10 cm to the CAX.

**Table 3:** Breast tangents eComp analysis data.

	*Statistics*	*MapCHECK vs. TPS calc.*	*PV unmodified vs. TPS pred.*	*PV modified vs. TPS pred.*	*EPIDose vs. TPS calc.*
2‐field plans	mean	99.1	83.8	90.1	99.4
(14 fields)	std dev	0.69	8.2	6.4	0.41
	min	97.8	81.1	76.7	98.6
	max	99.6	99.0	95.3	100.
3‐field plans	mean	97.5	78.2	98.1	96.0
(16 fields)	std dev	1.7	14.8	2.2	2.9
	min	92.8	41.0	90.0	91.8
	max	100.	95.2	99.7	99.7

Because the two EPID dosimetry systems are so different in their commissioning and calculation, it is difficult to isolate through the methods of this study the primary reason that Portal Dosimetry fails while EPIDose succeeds in accurately analyzing these off‐axis fields. However, three main differences should be emphasized. First, the Portal Dosimetry system compares a calibrated EPID image to a portal dose prediction algorithm (with known limitations^(^ ^24^ ^–^ ^26^ ^)^ that is separate from the TPS dose calculation algorithm, while the EPIDose system compares measurements converted to dose planes directly to planes calculated by the TPS dose calculation algorithm. Secondly, the Varian Portal Dosimetry system was not designed to be optimized by quantitative comparison to any other independent measuring device in the way that Sun Nuclear EPIDose was specifically developed to allow optimization of the dose conversion algorithm by quantitative comparison with MapCHECK. [Note: Previous studies have suggested methods to improve and tune the Varian Portal Dosimetry system, though all these methods essentially by‐pass or otherwise work around the vendor's suggested procedures.] Thirdly, the EPIDose system allows a correction for the EPID response to MLC transmitted radiation, though it is documented that the Varian system makes no such correction.^(^ ^26^ ^)^ However, in regard to this third point, it should be noted again that the MLC transmission correction of the EPIDose system does not affect fields of low modulation complexity (e.g., eComp breast tangents) to any large degree, since these fields are often closely equivalent to static MLC‐blocked fields for the majority of beam‐on time.

It should be pointed out that neither of the EPID dosimetry systems correct for backscatter from the imager arm. These complex effects are specific to both field size and position on the detecting surface, and may contribute dose errors of up to 2%–3% for flood‐field corrected images.^(^ ^32^ ^)^ Furthermore, it is also worth noting that the EPIDose optimization process relies on a diode array with poor data density, and so this process might benefit from comparison to some independent measurement modality with higher density (e.g., film or multiple merged MapCHECK acquisitions). This topic might warrant future investigation. For the purposes of this study, clinical cases were limited to the predominant photon beam of 6 M V. Since some clinical situations may require intensity modulation of a beam with higher energy, verification of EPIDose with such beams may warrant future investigation, as has been similarly published for Portal Dosimetry.^(^ ^24^ ^)^

### D. EPID VMAT QA

**Figure 6:**
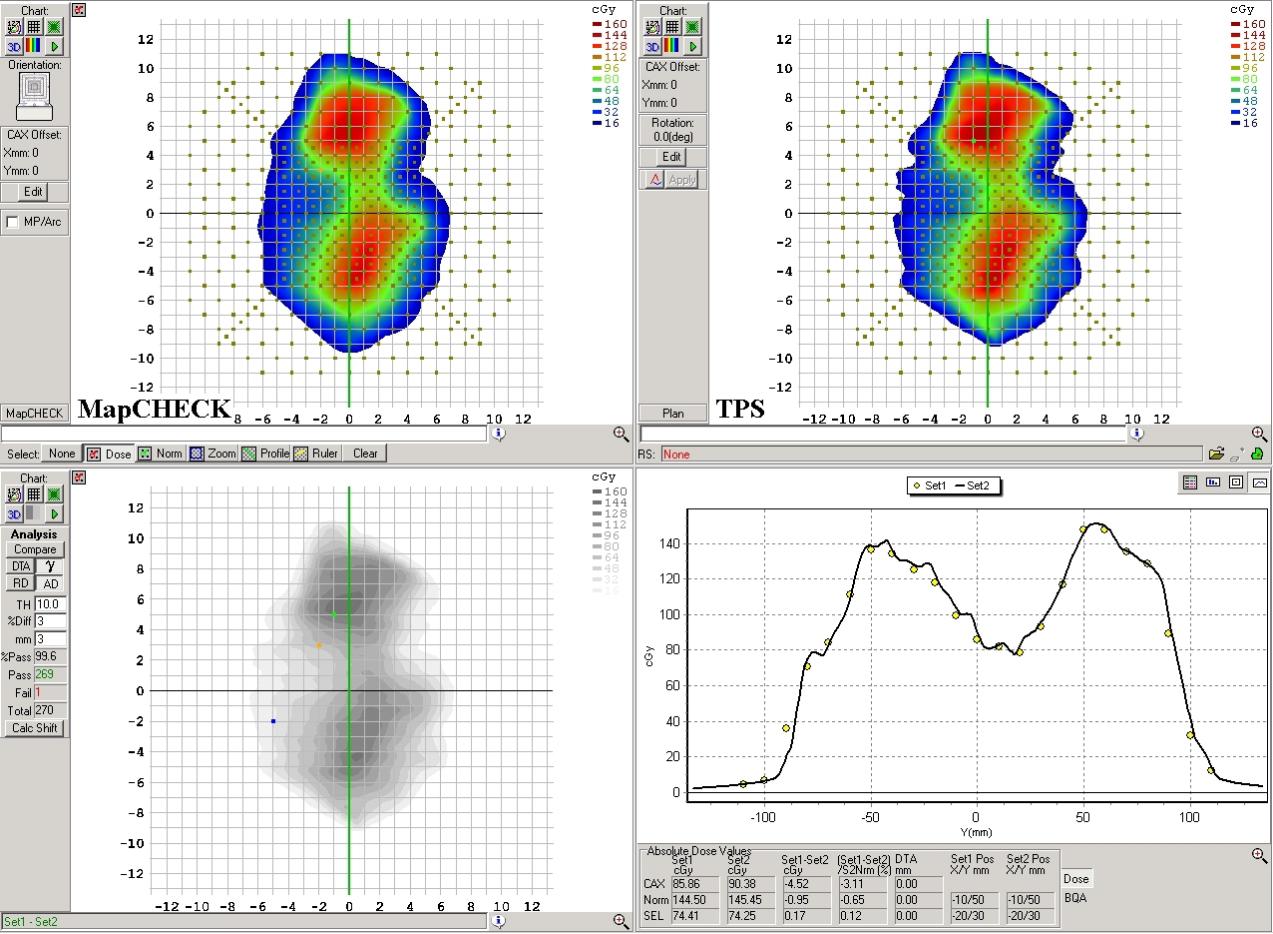
Sample H&N VMAT clinical case showing MapCHECK dose plane (top left), TPS calculated dose plane (top right), dose plane comparison showing 99.6% pass rate with gamma evaluation of 3%, 3 mm, and 10% threshold (bottom left), and a vertical dose line profile comparison between MapCHECK and TPS through the CAX (bottom right).

**Figure 7:**
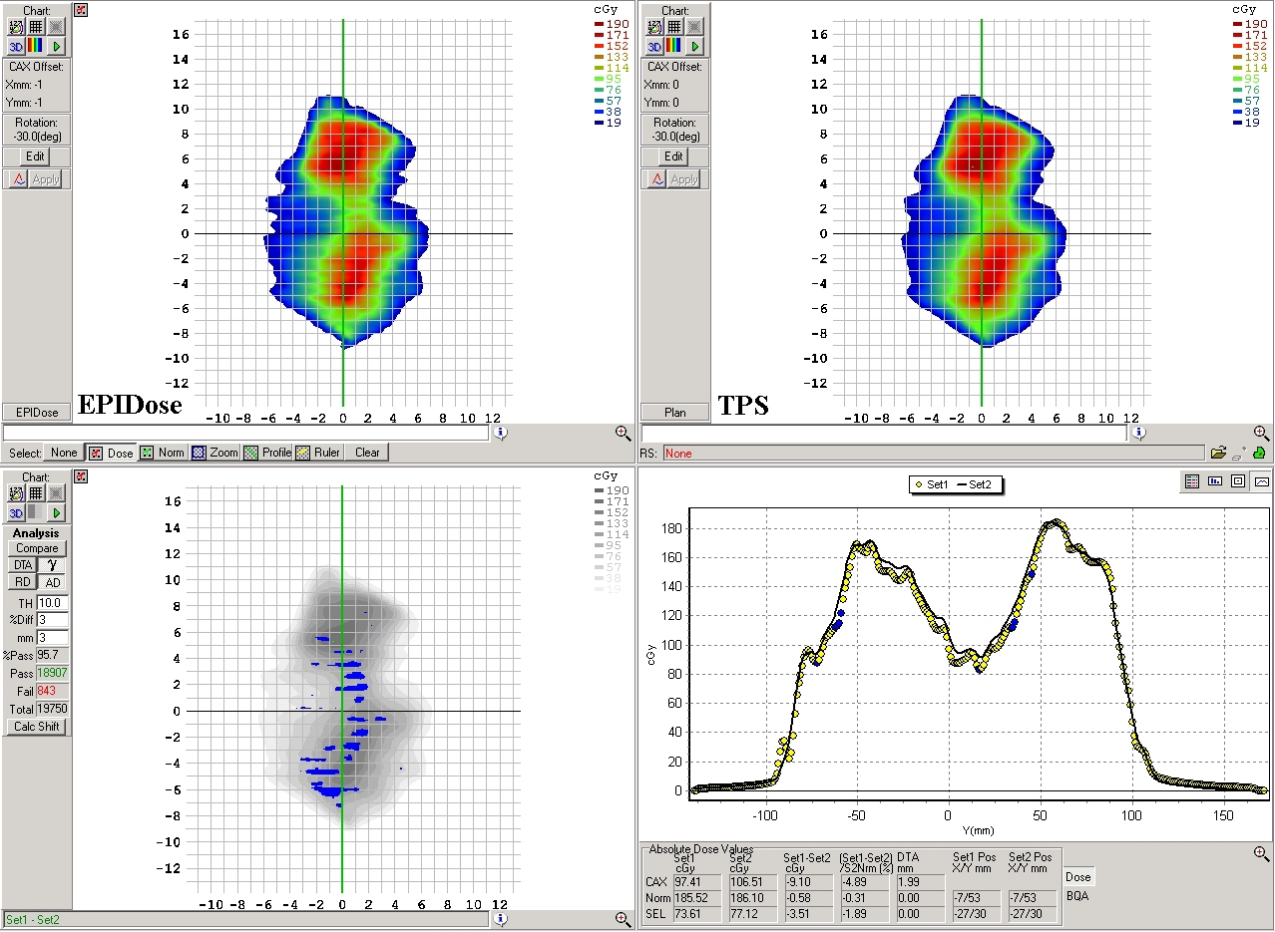
The same H&N VMAT clinical case as in Fig. 6, showing EPIDose dose plane (top left), TPS calculated dose plane (top right), dose plane comparison showing 95.7% pass rate with gamma evaluation of 3%, 3 mm, and 10% threshold (bottom left), and a vertical dose line profile comparison between EPIDose and TPS through the CAX (bottom right). High detector density of the EPID measurement identifies tongue‐and‐groove effects overlooked by the low‐density diode array measurement.

As for the VMAT cases, Table 4 lists the results for MapCHECK and EPIDose analysis of the 12 H&N and 14 prostate VMAT fields. Gamma evaluation of the MapCHECK and EPIDose measurements indicate very similar performance of these systems, within 1% as judged by the average of the individual pass rates (3%, 3 mm). Although PortalVision could be used to produce similar acquired images, at the time of this study (mid‐2010) Varian had not produced a commercial portal dosimetry prediction algorithm for RapidArc, though methods have been suggested in the literature.^(^ ^33^ ^,^ ^34^ ^)^ Figures 6 and 7 show the analysis of the same H&N fluence as acquired with the MapCHECK diode array and the EPIDose dosimetry system, respectively. With gamma evaluation of 3%, 3 mm, and 10% threshold, the MapCHECK produces a 99.6% pass rate for this fluence, with virtually no trouble areas illustrated in either the dose plane comparison (bottom left) or dose profile comparison (bottom right). Meanwhile, the gamma evaluation of the EPIDose dose plane produces a 95.7% pass rate, with clear tongue‐and‐groove effects apparent on the dose plane comparison, and corresponding cold sections in the dose profile comparison. The high data density of the EPIDose system makes it possible to expose these fine regions of error that the MapCHECK diode array does not detect.

**Table 4:** Prostate and H&N VMAT analysis data.

	*Statistics*	*MapCHECK (with IMF)*	*EPIDose*
Prostate	mean	98.0	98.2
(14 arcs)	std dev	1.89	1.65
	min	93.3	95.3
	max	100.	100.
H&N	mean	95.6	95.3
(12 arcs)	std dev	3.8	5.9
	min	86.8	84.8
	max	99.6	99.7

**Figure 8:**
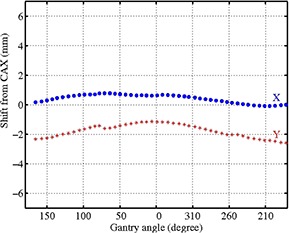
The cross‐plane (X) and in‐plane (Y) shifts of the EPID during 360° rotation, determined by examining the shift in isointensity lines from EPID images acquired at various gantry angles with a static square field.

The ability to perform pretreatment VMAT QA with an EPID is appealing both due to the high density of detectors and the ease of use and set up of the EPID (requiring no additional materials, cables, phantoms, etc.). From the results presented above, it appears that this EPID VMAT QA method provides good assurance that the treatment plan has been communicated accurately from the TPS to the record/verify system and on to the delivery system, and that the MLC motions are as planned. Still, there are inherent limitations with performing VMAT QA with a detector that is mounted to the rotating gantry, namely the lack of independent verification of gantry motion and the possibility of angle‐dependent detector sag due to gravitational force. As for the first concern, it is certain that with the adoption of any EPID dosimetry system for VMAT QA, there is an increased need for independent verification of gantry position and rotational speed^(^ ^35^ ^–^ ^38^ ^)^ on a per‐plan basis — possibly by inclinometer or examination of the linac log files. As for the second concern, the EPID certainly experiences gravitational conditions during VMAT delivery that differ from those present during the calibration process with fixed gantry.^(^ ^39^ ^)^ For the specific Varian linac/EPID system used in this study, we measured the EPID shift due to gantry rotation and found it to be reproducible with a magnitude of approximately 2 mm or less for all angles in both the in‐plane and cross‐plane directions (see Fig. 8), using the method proposed by Bakhtiari et al.^(^ ^40^ ^)^ Thus, in the case of this linac/EPID system, shifting due to gravitational force was not substantial enough to be deemed prohibitive for this study. However, EPID shifts due to gravitation may be quite different from machine to machine, and larger shifts may have a much more significant effect on VMAT QA results obtained by the methods discussed above. In the case of large shifts, it is difficult to conjecture a straightforward correction for images acquired via integrated acquisition mode since an entire rotation, requiring multiple shift corrections dependent on gantry angle, produces only one image. This issue calls for future investigation. While it is possible that the isocentric mounting of the diode array might exhibit similar sag, the method used in this study to quantify EPID sag is problematic for the MapCHECK/IMF combination simply due to the low data density of the diode array. The effect of gravitational force on isocentric mounting devices during rotational QA may also demand future investigation.

### E. Sensitivity to beam model error

Table 5 shows the IMRT QA results of five prostate fields before and after the MU/Gy error was introduced into the TPS beam model. Pass rates are calculated via gamma analysis of 3%, 3 mm, and 10% dose threshold. Since the PDIP algorithm does not audit the TPS calculation beyond the actual fluence, this error in the dose rate table calibration is not detected. However, similar QA and analysis with MapCHECK and EPIDose (measurements that are compared to dose planes calculated in water) reveal that there is a definite disagreement between TPS and field delivery.

**Table 5:** IMRT QA with MU/Gy TPS beam model error: individual pass rates with gamma evaluation of 3%, 3 mm, before and after the error was induced.

*Field*	*MapCHECK (before)*	*MapCHECK (after)*	*EPIDose (before)*	*EPIDose (after)*	*PV (before)*	*PV (after)*
1	98.6	45.3	97.7	49.3	97.8	98.6
2	100.	49.1	99.4	61.4	97.5	98.5
3	100.	52.6	99.4	50.7	98.9	98.9
4	100.	44.0	97.0	48.3	96.7	97.3
5	99.5	51.0	99.3	52.2	98.2	98.5
Average	99.6	48.5	98.6	52.4	97.8	98.4

Table 6 shows the IMRT QA results of 10 prostate fields before and after the 6 MV PBC model was altered by supplanting measured data with the Varian GBD. This error is more subtle than the previous example, and thus more sensitive criteria of 2%, 2 mm are used for the gamma evaluation. The EPIDose system proves most sensitive to this error, with an average decrease in pass rate of approximately 10%. Similar MapCHECK pass rates also decrease by over 3%, but the diode array is less sensitive to this subtle error due to its inferior data density,^(^ ^41^ ^)^ especially in the penumbra regions of IMRT fields where the induced errors are greatest.^(^ ^30^ ^)^ However, the Portal Dosimetry pass rates for these same fields change by less than 1% on average after the beam model error is induced. A small change is expected, since the portal dose prediction model is based on a different (i.e., GBD) intensity profile. However, this minor difference is not enough to expose the larger discrepancies in dose calculation caused by the differences in measured vs. GBD profiles. [Note: it should be remembered that the inherent flaw of the Varian GBD when combined with the PBC algorithm is only demonstrated by this data at the depth of EPIDose/MapCHECK measurements (i.e., 5 cm) and may result in larger or smaller magnitudes of error at other depths.]

**Table 6:** IMRT QA with profile/PDD TPS beam model error: individual pass rates with gamma evaluation of 2%, 2 mm, before and after the error was induced.

*Field*	*MapCHECK (before)*	*MapCHECK (after)*	*EPIDose (before)*	*EPIDose (after)*	*PV (before)*	*PV (after)*
1	97.4	93.5	92.2	83.2	96.5	96.3
2	98.2	94.7	96.2	90.0	98.9	97.6
3	97.8	91.5	96.3	88.7	99.1	98.5
4	98.6	98.0	90.2	73.7	96.8	96.3
5	94.4	91.6	90.9	77.2	98.2	98.3
6	97.4	94.8	92.5	84.0	98.7	97.2
7	97.3	96.2	89.2	75.3	97.9	97.1
8	98.6	95.6	95.3	86.8	98.5	97.1
9	94.6	93.6	94.0	86.7	98.6	97.2
10	99.4	96.5	94.1	82.3	98.5	97.2
Average	97.4	94.1	93.1	82.8	98.2	97.3

These results demonstrate the need for each user to understand the exact capabilities of the IMRT QA system of choice, such that limitations can be supplemented with other forms of QA. In its current form, Portal Dosimetry does not provide verification of the quality of the dose distribution planned for delivery to the patient. Thus, while the Portal Dosimetry system yields quantitative data to verify fluence calculation, data transfer, and fluence delivery, other forms of QA are necessary to audit the treatment planning system dose calculation models. In the two experimental examples above, the Portal Dosimetry system was tested for systematic errors in the beam model. However, it is important to remember that patient‐specific pretreatment verification is meant to test not only for systematic error, but also for random and unique problems. The two quantitative examples above stand as a proof of principle: errors that occur, either systematically or randomly, downstream from the actual fluence calculation may be masked by the Portal Dosimetry system's substitution of a portal dose prediction algorithm in the place of the actual dose calculation algorithm. Contrastingly, in addition to those checks provided by Portal Dosimetry, the EPIDose and MapCHECK systems provide an independent verification of dose calculation — albeit at a single depth, while recent studies suggest that this approach to dose verification may potentially overlook clinically significant errors.^(^ ^42^ ^–^ ^43^ ^)^ Further, for both arc and static‐gantry treatments, EPID treatment verification with any system is unable to audit dose perturbation due to the treatment couch, a limitation shared by phantom or array measurements with the gantry fixed at zero degrees.

## IV. CONCLUSIONS

Both the Portal Dosimetry and EPIDose algorithms yield similar pass rate results for the majority of clinical IMRT cases, though the pass rates generated by each system are not descriptive of the same information. EPIDose is able to accurately measure and analyze EPID response for off‐axis, asymmetric fields, while the Portal Dosimetry system is unable to adequately predict EPID response under similar conditions. The algorithms differ substantially in their comparison to the radiotherapy plan: EPIDose comparing calculated dose planes to the actual TPS dose calculation algorithm, and Portal Dosimetry comparing calibrated response planes to a TPS portal dose prediction algorithm based on the actual fluence calculation. Consequently, Portal Dosimetry does not directly audit the TPS dose calculation algorithm beyond the actual fluence, and other forms of QA (e.g., secondary MU calculations) are necessary to supplement this limitation. Further work is needed to determine what other beam model parameters might not be audited by the portal dose image prediction algorithm for IMRT QA. The EPIDose algorithm is able to accurately measure and analyze dose planes acquired from VMAT deliveries, within the limitations inherent to all methods of EPID rotational QA: the EPID is always orthogonal to the beam and may sag due to gravitational force. The high detector density and setup efficiency of EPIDs, combined with an accurate and reliable dosimetry algorithm, make electronic portal dosimetry an ideal alternative to more traditional, but less efficient, means of performing IMRT and VMAT planar QA.

## ACKNOWLEDGMENTS

The authors would like to thank Dr. Benjamin E. Nelms of Canis Lupus LLC (Merrimac, Wisconsin) for his valuable guidance in understanding and optimizing the EPIDose algorithm.
